# Oral Epithelial Remodeling Associated with Long-Term Contact with Conventional Coronal Dental Amalgam Restorations: A Retrospective Histopathological and Immunohistochemical Study

**DOI:** 10.3390/medicina62050963

**Published:** 2026-05-14

**Authors:** Roxana-Cristina Mehedinti, Dorin Ioan Cocoș, Ada Stefanescu, Madalina Nicoleta Matei, Gabriel Valeriu Popa, Dana Tutunaru

**Affiliations:** 1 Medical and Pharmaceutical Research Center, Faculty of Medicine and Pharmacy, “Dunărea de Jos” University of Galati, 800008 Galati, Romania; roxana.mehedinti@ugal.ro (R.-C.M.); madalina.matei@ugal.ro (M.N.M.); gabriel.popa@ugal.ro (G.V.P.); dana.tutunaru@ugal.ro (D.T.); 2 Dental-Medicine Department, Faculty of Medicine and Pharmacy, “Dunărea de Jos” University of Galati, 800201 Galati, Romania; 3 Clinical County Emergency Hospital “Sfantul Apostol Andrei”, 800578 Galati, Romania

**Keywords:** cytokeratin 19, oral mucosa, dental amalgam exposure, epithelial remodeling, chronic inflammation, Ki67, p53, immunohistochemistry

## Abstract

*Background and Objectives*: Prolonged contact between oral mucosa and dental amalgam restorations may influence local epithelial homeostasis, but the remodeling profile of clinically non-dysplastic mucosa exposed to long-standing amalgam remains insufficiently characterized. This study aimed to evaluate histopathological changes and CK19, Ki67, and p53 expression in the oral mucosa adjacent to long-term amalgam restorations. *Materials and Methods*: A retrospective observational analysis was performed on 108 oral mucosal specimens, including 78 samples in direct contact with amalgam restorations and 30 non-exposed controls. Exposed cases were grouped according to contact duration: 5–10 years, 11–20 years, and ≥21 years. Histopathological parameters and immunohistochemical expression of CK19, Ki67, and p53 were semi-quantitatively assessed, and an exploratory Integrated Epithelial Remodeling Score was calculated. *Results*: Longer amalgam exposure was significantly associated with increased inflammatory infiltrate, basal hyperplasia, acanthosis, fibrosis, suprabasal CK19 redistribution, and higher Ki67 labeling indices. The Integrated Epithelial Remodeling Score differed significantly among exposure groups, with higher values in intermediate- and long-duration exposure categories. p53 expression showed statistically detectable but heterogeneous variation. No epithelial dysplasia was observed. *Conclusions*: Long-term contact with dental amalgam restorations was associated with a coordinated, non-dysplastic remodeling phenotype of the oral mucosa. Given the age imbalance across exposure duration groups, these findings should be interpreted as exposure-associated patterns rather than evidence of a direct causal effect. Because no comparison group exposed to other restorative materials was included, material-specificity for dental amalgam cannot be inferred. In architecturally preserved mucosa, suprabasal CK19 expression may reflect adaptive epithelial plasticity rather than preneoplastic transformation.

## 1. Introduction

Dental amalgam has long been used in restorative dentistry because of its mechanical durability and clinical longevity, and in many patients, these restorations remain in close contact with the adjacent oral mucosa for extended periods [1,2]. Although the systemic safety of amalgam has been extensively discussed, comparatively less attention has been directed toward the local behavior of clinically non-neoplastic oral epithelium chronically exposed to amalgam-containing surfaces [3,4]. In this setting, the mucosa may be exposed to persistent low-intensity mechanical irritation and chemical stimulation, generating a local inflammatory microenvironment that can influence epithelial turnover and tissue homeostasis [5,6]. This concept is clinically relevant because dental restorative materials may participate in contact-associated mucosal reactions, including oral lichenoid lesions, in which chronic local inflammation and epithelial changes require careful clinicopathological interpretation [7,8,9,10].

Dental amalgam represents a heterogeneous group of mercury-based restorative alloys whose biological behavior may vary according to composition, copper content, corrosion resistance, surface aging, and clinical location [1,3,4]. Traditional low-copper and more recent high-copper formulations differ in their silver, tin, copper, and mercury-containing phases; although copper is an essential trace element involved in physiological redox regulation, excessive local exposure may contribute to oxidative stress and cytotoxicity, making the biological response concentration- and context-dependent [3,4,10]. In the oral environment, long-standing amalgam restorations are exposed to saliva, pH fluctuations, thermal variation, occlusal loading, biofilm accumulation, and surface wear, while cervical/proximal margins, roughened surfaces, and plaque-retentive areas may provide persistent mechanical, chemical, and inflammatory stimuli at the restoration–mucosa interface [2,4,5,7,11,12,13]. Although the use of dental amalgam has markedly declined in many European settings, including Romania, and is being progressively restricted in line with European mercury-reduction policies, many patients still retain restorations placed years or decades earlier; therefore, the current relevance of this topic lies in understanding the local mucosal response adjacent to pre-existing long-standing amalgam restorations rather than in supporting continued use of the material [1,2,14].

From a biological perspective, the oral mucosa should be interpreted not only as a passive lining tissue, but as an active epithelial barrier that integrates mechanical, microbial, chemical, and immune signals. Persistent local stimulation may alter barrier homeostasis by sustaining low-grade inflammation, modifying cytokine-mediated epithelial–stromal communication, and increasing the demand for basal cell renewal. In this context, epithelial remodeling represents a coordinated adaptive process involving proliferation, differentiation, cytoskeletal reorganization, and stromal matrix responses, rather than a purely morphologic observation. Recent reviews on oral mucosal biology, wound healing, anti-inflammatory mechanisms, and mucosal regeneration emphasize that inflammation, epithelial turnover, fibroblast activity, and extracellular matrix remodeling are functionally interconnected during mucosal adaptation and repair [8,9,10].

Oral stratified squamous epithelium is a dynamic barrier tissue capable of adapting to environmental stress through coordinated changes in cellular proliferation, differentiation, and cytoskeletal organization [11,12]. Therefore, microscopic epithelial remodeling may develop even in the absence of clinically evident lesions [13]. Such alterations should not be interpreted automatically as dysplastic change, particularly when epithelial architecture remains preserved, but may instead represent adaptive epithelial plasticity in response to chronic stimulation [13,14,15].

Cytokeratin 19 (CK19) is a low-molecular-weight intermediate filament protein that, in normal oral mucosa, is usually expressed mainly within the basal epithelial compartment [16]. Extension of CK19 staining into suprabasal epithelial layers has been reported in oral epithelial dysplasia and malignant transformation, where it is commonly regarded as an indicator of disturbed epithelial differentiation [17,18]. Nevertheless, the significance of CK19 redistribution in chronically irritated but morphologically non-neoplastic mucosa remains incompletely understood [19]. Interpreting CK19 expression almost exclusively within a preneoplastic framework may therefore overlook its possible involvement in reactive or adaptive epithelial remodeling [20]. In parallel, Ki67 immunolabeling offers an estimate of epithelial proliferative activity and turnover, whereas p53 expression may indicate cellular stress and checkpoint-related responses [21,22]. The biological meaning of these markers in non-dysplastic mucosa depends on their relationship with epithelial architecture, inflammatory context, and preservation of tissue organization.

The present study was designed to assess histopathological and immunohistochemical changes in the oral mucosa located adjacent to long-standing dental amalgam restorations. A control group of oral mucosal specimens without direct amalgam contact was included to separate baseline mucosal variability from changes potentially associated with chronic exposure. In addition to evaluating individual histological and immunohistochemical parameters, we developed an exploratory Integrated Epithelial Remodeling Score (IERS) to determine whether structural epithelial changes, inflammatory intensity, CK19 redistribution, and proliferative activity form a coordinated remodeling profile related to exposure duration. We hypothesized that long-standing contact between oral mucosa and conventional coronal dental amalgam restorations is associated with a coordinated, non-dysplastic epithelial remodeling phenotype characterized by inflammatory activation, basal epithelial expansion, CK19 redistribution, and increased Ki67-related proliferative activity.

## 2. Materials and Methods

### 2.1. Study Design, Setting, Patients, and Tissue Sampling

This retrospective observational study included 108 unique patients, corresponding to 108 formalin-fixed, paraffin-embedded oral mucosal specimens retrieved from the archives of the Department of Oral Pathology over a 4-year retrospective period, from April 2022 to March 2026. The clinical procedures were performed in the affiliated dental clinical setting of the Faculty of Medicine and Pharmacy, “Dunărea de Jos” University of Galati, Romania, and histopathological and immunohistochemical analyses were carried out at the Department of Oral Pathology of the same institution. The study was conducted in accordance with the Declaration of Helsinki and was approved by the Ethics Committee of the University Center of Dental Medicine of Galati, “Dunărea de Jos” University of Galati, Romania (approval no. 16/23 April2026). Written informed consent for the diagnostic procedures and for the use of biological material for research purposes was obtained from all included patients.

The study cohort included 78 specimens obtained from mucosa directly adjacent to long-standing conventional coronal dental amalgam restorations and 30 control specimens from oral mucosa without direct contact with amalgam restorations. Exposed cases were classified according to the documented duration of mucosal contact with amalgam restorations into three predefined groups: 5–10 years, 11–20 years, and ≥21 years. The overall cohort included 63 females and 45 males. Sex distribution by exposure duration group is reported in Section 3. Smoking status was ascertained from the available clinical records and preoperative anamnesis forms completed at the time of dental/oral surgical evaluation. Patients were classified as non-smokers when no active smoking habit was documented in the medical history. Because of the retrospective design, no biochemical verification of smoking exposure, such as cotinine testing or exhaled carbon monoxide assessment, was available. Data regarding frequent spicy food consumption were not consistently available in the retrospective records and were therefore not included as a comparative variable; this limitation was considered when interpreting potential lifestyle-related modifiers of mucosal inflammation.

All mucosal specimens were obtained during alveoloplastic extractions performed for fractured teeth considered non-restorable by conventional restorative or prosthetic means. During alveoloplasty, gingival or alveolar mucosal tissue that would otherwise have been discarded as surgical excess tissue was preserved for histopathological and immunohistochemical assessment within the framework of a broader research project on local mucosal responses adjacent to dental restorations. No mucosal specimens were collected during simple extractions without alveoloplasty, and tissue sampling was not performed because of clinical suspicion of epithelial dysplasia, oral potentially malignant disorder, or carcinoma.

Control specimens were obtained from similar alveoloplastic extraction procedures in patients without direct mucosal contact with amalgam restorations and without clinical or histopathological evidence of inflammatory, dysplastic, or neoplastic mucosal alterations. Whenever possible, control tissues were selected from anatomically comparable gingival or alveolar mucosal sites to reduce site-related variability.

In the exposed group, amalgam exposure referred exclusively to conventional coronal amalgam restorations located adjacent to the sampled mucosa. Retrograde amalgam fillings placed after apicoectomy were not included. The restorations were predominantly cervical and proximal coronal amalgam restorations, corresponding mainly to class V and class II restorations, in which restoration margins or adjacent surfaces were in close proximity to the gingival or oral mucosa. Cases were included only when clinical records indicated long-standing mucosal adjacency or direct contact with the amalgam-restored tooth surface. Detailed quantitative measurements of restoration surface area, marginal roughness, exact contact pressure, plaque retention, and standardized topographic contact patterns were not consistently available in the retrospective records.

Tissue sampling was performed by the attending oral/dento-alveolar surgeon during the indicated surgical procedure. Immediately after excision, specimens were fixed in 10% neutral-buffered formalin, processed routinely, and embedded in paraffin. Histopathological evaluation and immunohistochemical scoring were performed independently by two experienced pathologists at the Department of Oral Pathology, Faculty of Medicine and Pharmacy, “Dunărea de Jos” University of Galati, Romania, and discrepancies were resolved by consensus review.

### 2.2. Histopathological Evaluation

For histopathological assessment, 4 μm sections were cut from paraffin blocks and stained with hematoxylin–eosin (H&E; Bio-Optica, Milan, Italy). Slides were evaluated using a predefined semi-quantitative scoring system, with discrepant assessments resolved by consensus review. A semi-quantitative scoring system was applied to evaluate epithelial and stromal parameters. Basal hyperplasia was graded as: 0—absent, 1—mild, 2—moderate, 3—marked, based on the degree of basal layer expansion and elongation of epithelial ridges. Acanthosis was graded on a similar four-point scale (0–3) according to the thickness of the spinous layer. Parakeratosis was recorded as: 0—absent, 1—present. Spongiosis was graded from 0 to 3 according to the extent of intercellular edema. Subepithelial fibrosis was evaluated on a four-point scale (0–3) reflecting the density and extent of collagen deposition beneath the epithelial layer. The inflammatory infiltrate was graded as: 1—mild and focal, 2—moderate and diffuse, 3—intense.

### 2.3. Immunohistochemistry

Immunohistochemical staining was performed on 4 μm sections mounted on positively charged slides (Thermo Fisher Scientific, Waltham, MA, USA). After deparaffinization in xylene (Merck KGaA, Darmstadt, Germany) and rehydration through graded alcohol solutions (Merck KGaA, Darmstadt, Germany), antigen retrieval was carried out using heat-induced epitope retrieval. Citrate buffer (pH 6.0; Agilent Technologies, Santa Clara, CA, USA) was used for cytokeratin 19 (CK19) and Ki67. EDTA buffer (pH 9.0; Agilent Technologies, Santa Clara, CA, USA) was used for p53. Endogenous peroxidase activity was blocked by incubation with 3% hydrogen peroxide (Sigma-Aldrich, St. Louis, MO, USA). Sections were incubated with primary antibodies against: CK19 (mouse monoclonal antibody, clone RCK108; Agilent Technologies, Santa Clara, CA, USA), Ki67 (mouse monoclonal antibody, clone MIB-1; Dako, Glostrup, Denmark), and p53 (mouse monoclonal antibody, clone DO-7; Dako, Glostrup, Denmark), using manufacturer-recommended dilutions and incubation times. Immunodetection was performed using a peroxidase detection system (EnVision FLEX Detection System; Dako, Glostrup, Denmark), with 3,3′-diaminobenzidine (DAB; Dako, Glostrup, Denmark) as chromogen. Slides were counterstained with hematoxylin (Merck KGaA, Darmstadt, Germany), dehydrated, cleared, and mounted (DPX mounting medium, Sigma-Aldrich, St. Louis, MO, USA). Appropriate positive and negative tissue controls were included for each antibody.

#### Immunohistochemical Scoring

CK19 expression was evaluated semi-quantitatively according to the extent of epithelial distribution and staining intensity: score 0—absence of staining, score 1—weak staining confined to basal/parabasal layers, score 2—moderate staining extending into suprabasal layers, score 3—strong staining involving most or the entire epithelial thickness.

The Ki67 labeling index was determined by counting at least 300 epithelial cells in areas with the highest proliferative activity (hot spots). The percentage of positively stained nuclei was categorized as: Score 0—≤5%, score 1—6–10%, score 2—≥11%.

For p53, only nuclear staining was considered positive. Expression was categorized into three groups: Score 0—absence of staining or <5% positive cells, score 1—weak nuclear staining in 6–10% of epithelial cells, score 2—moderate or strong nuclear staining in ≥11% of epithelial cells. In the absence of morphological features of epithelial dysplasia or carcinoma, lack of p53 staining was interpreted as physiological baseline expression rather than a null-type mutation pattern. The study did not aim to infer *TP53* mutational status but rather to assess relative variation in protein expression within a reactive inflammatory context.

### 2.4. Construction of the Integrated Epithelial Remodeling Score (IERS)

To evaluate the cumulative burden of structural and proliferative alterations at the individual case level, an Integrated Epithelial Remodeling Score (IERS) was constructed. The score represents the sum of four parameters that demonstrated significant associations with exposure duration: basal hyperplasia (0–3), inflammatory infiltrate (1–3), CK19 redistribution (0–3), and categorized Ki67 proliferative index (0–2). The theoretical IERS range extended from 1 to 11, with higher values reflecting greater epithelial remodeling intensity. In the present dataset, inflammatory infiltrate scores ranged from 1 to 3, therefore the lowest possible composite score was 1. p53 expression was not included in the composite score due to its weaker and statistically non-independent association with exposure category. IERS was analyzed as a continuous variable representing cumulative remodeling intensity across structural, inflammatory, and proliferative parameters.

### 2.5. Statistical Analysis

Statistical analysis was performed using GraphPad Prism version 9.0 (GraphPad Software, San Diego, CA, USA) and IBM SPSS Statistics version 26.0 (IBM Corp., Armonk, NY, USA), as appropriate. Continuous variables were expressed as mean ± standard deviation, whereas ordinal variables were summarized using medians and interquartile ranges. Normality was assessed for continuous variables using the Shapiro–Wilk test and visual inspection of distribution plots. Normality testing was not applied to histopathological and immunohistochemical scores, because these variables were ordinal by design nd were therefore analyzed using non-parametric methods irrespective of distributional assumptions.

Intergroup comparisons of continuous variables were performed using one-way analysis of variance or the Kruskal–Wallis test, depending on distributional characteristics. Ordinal histopathological and immunohistochemical scores were compared across exposure categories using the Kruskal–Wallis test. When significant intergroup differences were detected, post hoc pairwise comparisons were performed using Dunn’s test with Bonferroni correction. Categorical variables were compared using the chi-square test or Fisher’s exact test, as appropriate. Associations between exposure duration, age, histopathological parameters, immunohistochemical markers, and the composite IERS were evaluated using Spearman’s rank correlation coefficient.

To address the potential confounding effect of age, age-adjusted multivariable analyses were performed. The association between the exposure duration category and IERS was assessed using multivariable linear regression, with age included as a covariate. For the main remodeling parameters, binary logistic regression was performed using clinically relevant high-grade thresholds, including inflammatory infiltrate ≥ 2, basal hyperplasia ≥ 2, suprabasal CK19 expression ≥ 2, and Ki67 score 2. The exposure duration category and age were entered simultaneously as predictors in each model. Age-matching was not performed because of the retrospective archival design and the limited availability of eligible control specimens fulfilling the same sampling criteria, including alveoloplastic extraction, absence of direct amalgam contact, absence of clinically evident mucosal disease, and histopathologically preserved non-dysplastic mucosa. Restricting the control group to older patients only would have substantially reduced the available control sample and could have introduced additional selection bias. Therefore, age was handled statistically as a covariate in multivariable models, while residual confounding by age was explicitly considered in the interpretation of the findings. A two-tailed *p*-value < 0.05 was considered statistically significant.

## 3. Results

### 3.1. Study Cohort and Demographic Characteristics

The clinicodemographic characteristics of the study cohort are summarized in Table 1. All included patients were classified as non-smokers based on available clinical records and preoperative anamnesis forms; however, no biochemical verification of smoking exposure was available. Data regarding frequent spicy food consumption were not consistently available in the retrospective records and were therefore not included as a comparative variable. All samples were obtained as excess gingival or alveolar mucosal tissue during alveoloplastic extractions of fractured non-restorable teeth. Exposed specimens were collected adjacent to conventional coronal amalgam restorations, whereas control specimens were obtained from comparable alveoloplastic procedures without direct amalgam contact. A significant difference in age distribution was observed among the study groups (*p* < 0.001), consistent with the expected association between longer restoration retention and increasing patient age. Because age may act as a potential confounding variable in long-duration exposure studies, this difference was considered during the interpretation of exposure-related remodeling patterns and was further addressed by age-adjusted analyses. Sex distribution did not differ significantly across exposure categories (*p* = 0.084).

### 3.2. Histopathological Alterations Across Control and Exposure Groups

Comparative histopathological evaluation across the four study groups revealed an ordered pattern of epithelial (basal hyperplasia, acanthosis, parakeratosis, spongiosis) and stromal remodeling (subepithelial fibrosis, inflammatory infiltrate) observed across increasing categories of amalgam exposure duration. The inclusion of a control group enabled clear differentiation between baseline mucosal morphology and exposure-associated structural alterations. Basal epithelial hyperplasia demonstrated significant intergroup variation (*p* = 0.004). When moderate-to-marked changes (scores 2–3) were considered, their prevalence increased from 25.0% in the 5–10-year group to 61.8% in the 11–20-year group and 75.0% in specimens exceeding 21 years of exposure. This distribution was consistent with progressive expansion of the basal proliferative compartment across longer exposure duration categories. Acanthosis also varied significantly across groups (*p* = 0.003). Moderate-to-marked acanthosis (scores 2–3) was observed in 30.0% of controls and 35.7% of the 5–10-year group, increasing to 67.6% in the 11–20-year category and 87.5% in the ≥21-year group. These findings support a structured thickening of the spinous layer observed in the longer exposure duration groups.

Parakeratosis showed a statistically significant but less pronounced intergroup difference (*p* = 0.042). The proportion of specimens exhibiting parakeratosis rose from 6.7% in controls to 42.9% in the 5–10-year group, 52.9% in the 11–20-year group, and 56.3% in the ≥21-year group. Although statistically detectable, this parameter displayed greater variability and a less strictly ordered gradient compared with other remodeling features. Spongiosis demonstrated significant variation among groups (*p* = 0.017). Moderate-to-marked intercellular edema (scores 2–3) was present in 20.0% of control specimens and 21.4% of the 5–10-year group, increasing to 44.1% in the 11–20-year group and 62.5% in the ≥21-year group. This trend suggests a progressive alteration of epithelial intercellular cohesion across longer exposure duration categories. Subepithelial fibrosis exhibited a marked exposure-associated distribution (*p* < 0.001). Moderate-to-severe fibrosis (scores 2–3) was identified in 30.0% of controls and 39.3% of the 5–10-year group, rising to 73.5% in the 11–20-year group and 87.5% in the longest exposure category. Collagen deposition beneath the epithelial basement membrane appeared progressively denser and more extensively organized with increasing duration of amalgam contact.

Inflammatory infiltrate displayed the strongest exposure-related gradient (*p* < 0.001). Moderate-to-intense inflammatory infiltration (scores 2–3) was observed in 30.0% of control tissues and 32.1% of the 5–10-year group, increasing substantially to 67.6% in the 11–20-year group and 87.5% in specimens exceeding 21 years of exposure. The inflammatory escalation closely paralleled epithelial thickening and stromal fibrosis, indicating coordinated remodeling within a chronically stimulated mucosal microenvironment (Table 2).

**Figure 1:**
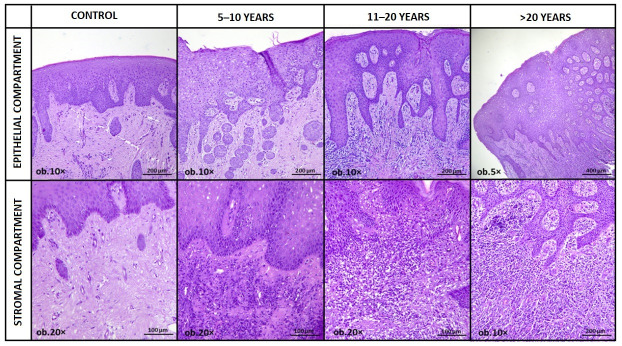
Representative hematoxylin-eosin micrographs illustrating progressive epithelial and stromal remodeling across increasing durations of amalgam exposure.

Taken together, these histopathological findings delineate a coherent remodeling sequence. Control mucosa maintains a relatively stable epithelial architecture with limited inflammatory activity and minimal stromal alteration. Across increasing exposure duration categories, a structured escalation was observed, characterized by inflammatory amplification, basal compartment expansion, progressive epithelial thickening, intercellular edema, and cumulative stromal fibrosis. Importantly, these changes evolve gradually across ordered exposure categories and do not display abrupt transitions or architectural features suggestive of epithelial dysplasia. This ordered progression supports the interpretation of cumulative epithelial adaptation within a chronic inflammatory context rather than isolated or randomly distributed histological variability (Figure 1).

### 3.3. Immunohistochemical Expression Patterns Across Control and Exposure Groups

**Figure 2:**
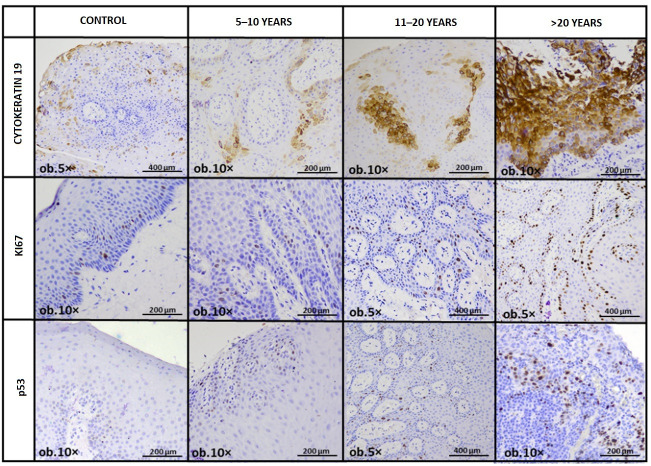
Representative immunohistochemical expression of CK19, Ki67, and p53 across control and amalgam-exposed oral mucosal specimens. Images illustrate staining patterns in the control group and in the 5–10-year, 11–20-year, and ≥21-year exposure groups.

Immunohistochemical evaluation revealed a structured redistribution of epithelial biomarkers paralleling the histopathological remodeling observed across exposure categories (Figure 2).

When control and exposed specimens were analysed together, significant intergroup differences emerged for CK19 and Ki67, while p53 demonstrated a more heterogeneous pattern. CK19 expression differed significantly among the four groups (*p* < 0.001). In control mucosa, immunoreactivity was predominantly restricted to the basal and parabasal layers, with 83.3% of cases showing low-level expression (scores 0–1: 30.0% score 0; 53.3% score 1). Suprabasal extension (scores 2–3) was observed in only 16.7% of controls. In the 5–10-year group, a comparable distribution persisted, with 75.0% of cases remaining within scores 0–1 and 25.0% exhibiting suprabasal extension. A marked shift became evident in the 11–20-year group, where suprabasal CK19 expression (scores 2–3) increased to 55.9% of cases. The most pronounced redistribution was observed in the ≥21-year category, in which 87.5% of specimens demonstrated extended suprabasal staining, including 56.3% with near full-thickness involvement (score 3). This progressive escalation was consistent with the expansion of a CK19-positive epithelial compartment across longer exposure duration categories.

Ki67 proliferative activity followed a similarly structured gradient (*p* < 0.001). In control tissues, low labeling indices predominated: 86.7% of cases fell within scores 0–1 (26.7% score 0; 60.0% score 1), and only 13.3% exhibited elevated proliferative activity (score 2). The 5–10-year group demonstrated a comparable profile, with 85.7% of cases remaining within low proliferative categories and 14.3% showing increased labeling. A substantial upward shift occurred in the 11–20-year group, where high proliferative indices (score 2) were observed in 64.7% of cases. In the ≥21-year group, elevated Ki67 expression was present in 87.5% of specimens, indicating a pronounced expansion of the proliferative compartment. Importantly, Ki67-positive nuclei remained largely concentrated within expanded basal and suprabasal compartments rather than diffusely distributed, supporting a reactive proliferative pattern rather than architectural disorganization.

Protein p53 expression demonstrated greater variability across groups (*p* = 0.023) and did not exhibit the same orderly stepwise gradient observed for CK19 and Ki67. In the control mucosa, staining was absent or limited in the majority of cases, with 66.7% of specimens showing score 0, 23.3% score 1, and only 6.7% score 2 expression. In the 5–10-year group, moderate expression (score 1) was observed in 21.4% of cases and higher expression (score 2) in 7.1%. In the 11–20-year category, score 2 expression increased to 17.6%, while in the ≥21-year group it reached 37.5%. Despite this numerical increase in higher categories, the overall distribution lacked the consistent stepwise escalation pattern seen in CK19 and Ki67, and strong diffuse overexpression was not observed (Table 3).

**Figure 3:**
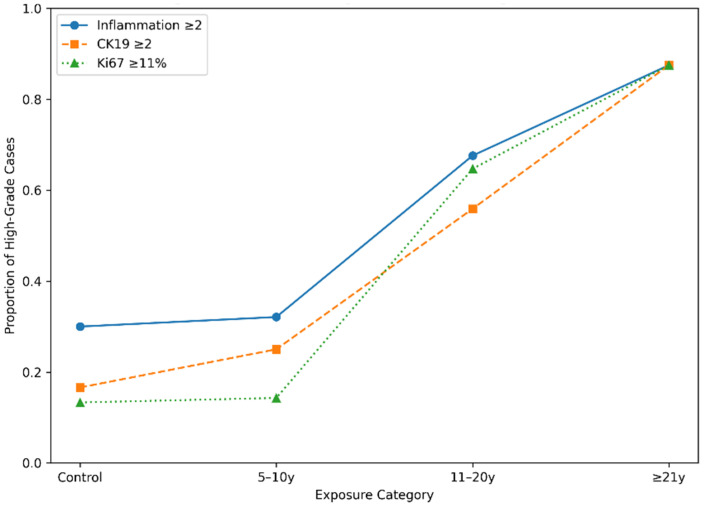
Distribution of high-grade remodeling parameters according to amalgam exposure duration. The graph illustrates the proportion of cases with inflammatory infiltrate ≥ 2, suprabasal CK19 extension ≥ 2, and Ki67 labeling index ≥ 11% across the control, 5–10-year, 11–20-year, and ≥21-year groups.

Collectively, the immunophenotypic findings align with the histopathological observations. Progressive suprabasal CK19 redistribution and increasing Ki67 proliferative activity were observed across longer exposure duration categories, whereas p53 modulation, although still statistically significant, appeared more variable and did not demonstrate the same ordered gradient. This pattern supports coordinated epithelial remodeling with reactive proliferative reinforcement rather than dysplastic transformation (Figure 3).

### 3.4. Integrated Epithelial Remodeling Score (IERS)

**Figure 4:**
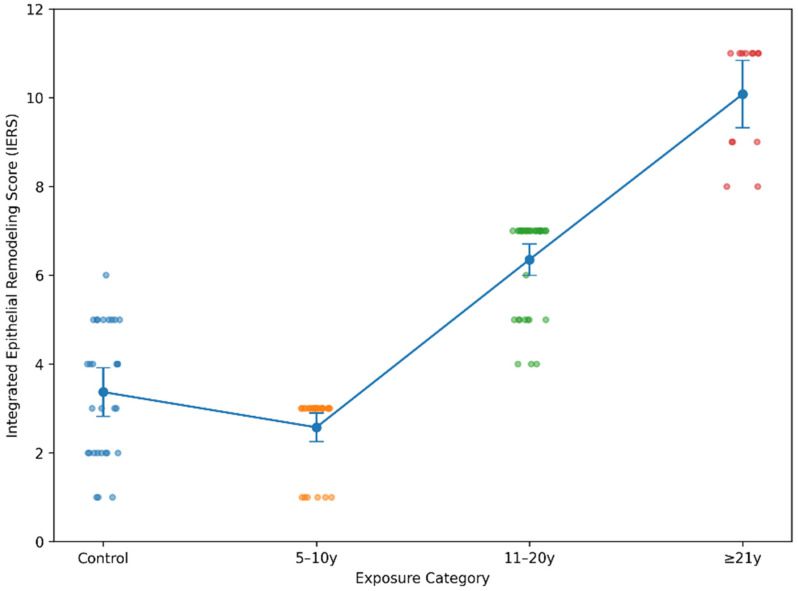
Distribution of the Integrated Epithelial Remodeling Score (IERS) according to amalgam exposure duration. Individual values, group means, and 95% confidence intervals are shown.

In order to capture the cumulative burden of epithelial and inflammatory alterations at the individual case level, an Integrated Epithelial Remodeling Score (IERS) was calculated for each specimen. The score incorporated four parameters demonstrating structured intergroup variation: basal hyperplasia, inflammatory infiltrate intensity, CK19 redistribution, and categorized Ki67 proliferative index. By integrating structural and functional components into a composite metric, IERS provided a global representation of remodeling intensity rather than isolated marker fluctuations. When analyzed across the four study groups, IERS demonstrated significant intergroup differences (*p* < 0.001). Control specimens exhibited low composite scores, consistent with limited basal expansion, minimal inflammatory infiltration, restricted CK19 distribution, and low proliferative activity. Interestingly, the 5–10-year exposure group showed comparable or slightly lower mean IERS values, reflecting early-stage variability and partial overlap with baseline mucosal architecture. A marked upward shift became evident in the 11–20-year category, where composite scores were substantially elevated compared to both control and short-term exposure groups. The ≥21-year group exhibited the highest IERS values, with clustering toward the upper range of the score spectrum, indicating cumulative reinforcement of epithelial structural and proliferative alterations (Figure 4).

Spearman’s rank correlation analysis confirmed a strong positive association between exposure duration category and IERS (*p* < 0.001), supporting a duration-associated intensification of remodeling despite early non-linear variability. Although interindividual heterogeneity was present within each exposure category, the overall distribution pattern revealed a clear escalation in intermediate and long-term exposure groups. Collectively, these findings indicate that epithelial remodeling was not distributed as an abrupt or dichotomous state, but rather showed a progressive, threshold-like intensification in the intermediate and long-duration exposure groups.

### 3.5. Correlation Network of Structural, Inflammatory, and Proliferative Parameters

To further elucidate the internal structure of the remodeling phenotype, a comprehensive Spearman correlation analysis was performed across exposure duration, age, histopathological parameters, immunohistochemical markers, and the composite IERS. The resulting matrix revealed a hierarchically organized interaction network linking inflammatory escalation, epithelial structural expansion, cytokeratin redistribution, proliferative amplification, and cumulative remodeling burden.

Exposure category demonstrated strong positive correlations with the principal remodeling parameters. The association with inflammatory infiltrate was particularly pronounced (*p* < 0.001), indicating that longer duration of amalgam contact closely paralleled increasing inflammatory intensity. A similarly strong correlation was observed between exposure category and Ki67 proliferative index (*p* < 0.001), reflecting progressive amplification of epithelial proliferative activity. Basal hyperplasia (*p* < 0.001), CK19 redistribution (*p* < 0.001), and subepithelial fibrosis (*p* < 0.001) also demonstrated significant positive associations with exposure duration, supporting the presence of coordinated epithelial-stromal adaptation. The composite IERS exhibited a particularly strong correlation with exposure category (*p* < 0.001), supporting the presence of progressively higher cumulative remodeling intensity across longer exposure duration categories. This coefficient ranked among the highest within the correlation matrix, reinforcing the ordered exposure-remodeling relationship observed in group comparisons. Age demonstrated moderate positive correlations with inflammatory infiltrate, basal hyperplasia, Ki67 category, CK19 redistribution, and IERS (*p* < 0.001). Although statistically significant, these coefficients were consistently lower than those observed for exposure category in the key remodeling parameters, suggesting that chronological aging contributes to variability but does not fully account for the structured exposure-associated gradient. Within the histopathological variables themselves, strong interrelationships were observed. Basal hyperplasia and inflammatory infiltrate were tightly linked, indicating that basal compartment expansion occurs within an inflammatory microenvironment. Basal hyperplasia also correlated strongly with CK19 redistribution and with subepithelial fibrosis, supporting parallel epithelial and stromal remodeling (*p* < 0.001).

**Figure 5:**
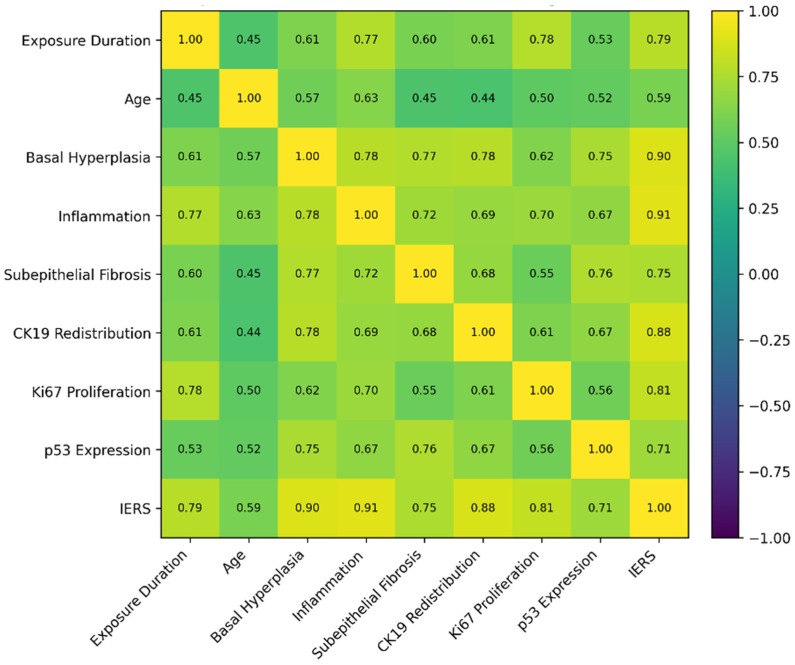
Spearman correlation matrix of clinical, histopathological, and immunohistochemical parameters.

Inflammatory infiltrate correlated positively with CK19 redistribution and Ki67 category, reinforcing the coupling between inflammatory signaling and proliferative reinforcement (*p* < 0.001). CK19 redistribution itself was strongly associated with IERS, while Ki67 category demonstrated a robust correlation with the composite score (*p* < 0.001). These high coefficients confirm that cytokeratin redistribution and proliferative amplification are integral components of cumulative remodeling intensity. p53 expression displayed moderate positive correlations with basal hyperplasia, fibrosis, inflammatory infiltrate, and CK19 redistribution. Although statistically significant, these associations did not exceed those observed for the CK19–Ki67 axis and did not demonstrate a dominant hierarchical position within the network (Figure 5).

### 3.6. Age-Adjusted Analysis

Because age differed significantly across exposure duration groups, age-adjusted analyses were performed to evaluate whether exposure duration remained associated with the main remodeling outcomes. In multivariable linear regression including exposure duration category and age as predictors, exposure duration remained associated with IERS after adjustment for age (β = 1.12, 95% CI: 0.70–1.54, *p* < 0.001), whereas age showed only a weaker borderline association with IERS (β = 0.03, 95% CI: −0.00–0.08, *p* = 0.061).

Age-adjusted binary logistic regression analyses showed that longer exposure duration remained associated with higher odds of high-grade inflammatory infiltrate (aOR = 2.08, 95% CI: 1.28–3.39, *p* = 0.003), basal hyperplasia (aOR = 1.82, 95% CI: 1.14–2.91, *p* = 0.012), suprabasal CK19 redistribution (aOR = 2.35, 95% CI: 1.40–3.95, *p* = 0.001), and elevated Ki67 expression (aOR = 2.51, 95% CI: 1.46–4.31, *p* < 0.001). In the same models, age showed weak and non-significant associations with high-grade inflammatory infiltrate (aOR = 1.03, 95% CI: 0.99–1.08, *p* = 0.119), basal hyperplasia (aOR = 1.04, 95% CI: 0.99–1.09, *p* = 0.087), CK19 redistribution (aOR = 1.02, 95% CI: 0.98–1.07, *p* = 0.241), and elevated Ki67 expression (aOR = 1.03, 95% CI: 0.98–1.08, *p* = 0.168). The full age-adjusted regression results are summarized in Table 4. These findings suggest that the exposure-associated remodeling pattern was not fully explained by chronological age alone. However, given the retrospective design and age imbalance among exposure groups, residual confounding cannot be excluded.

## 4. Discussion

The present study identifies an exposure-associated pattern of epithelial remodeling in oral mucosa adjacent to long-standing conventional coronal dental amalgam restorations. However, because longer exposure duration was accompanied by higher patient age, these findings should be interpreted cautiously and should not be regarded as proof of a direct causal effect. Rather than showing unrelated changes in individual markers, the findings describe an integrated remodeling profile in which inflammatory intensification, expansion of the basal epithelial compartment, cytokeratin redistribution, and increased proliferative activity occur in parallel. Importantly, these alterations were observed in morphologically preserved epithelium and in the absence of dysplasia, supporting their interpretation as regulated adaptive plasticity rather than early neoplastic transformation.

This interpretation is also clinically relevant because contact-associated oral mucosal reactions, including lichenoid lesions, may present with inflammatory and epithelial alterations that require contextual histopathological interpretation [7]. The biological relevance of this remodeling pattern lies in the role of the oral epithelium as a dynamic barrier interface. Chronic mucosal inflammation may increase epithelial turnover through cytokine-mediated activation of basal and parabasal compartments, while sustained epithelial–stromal communication can contribute to acanthosis, fibrosis, and altered cytokeratin expression. Therefore, the simultaneous increase in inflammatory infiltrate, basal hyperplasia, Ki67 expression, and suprabasal CK19 redistribution suggests a coordinated barrier-adaptation response rather than isolated biomarker variation. This interpretation is consistent with contemporary models of oral mucosal repair and inflammatory remodeling, in which epithelial proliferation, cytoskeletal plasticity, fibroblast activity, and connective-tissue remodeling are functionally linked during persistent tissue stress [8,9,10].

Several mechanisms may plausibly explain how long-standing amalgam restorations could participate in local mucosal stimulation. Dental restorations are exposed to a complex oral environment characterized by saliva, pH fluctuations, thermal changes, occlusal loading, microbial biofilms, and long-term functional wear, all of which may influence restoration aging and biological interaction with adjacent tissues [2,4]. In the case of dental amalgam, mercury-containing phases and alloy components such as silver, copper, and tin have been discussed in relation to material biocompatibility and potential local or systemic biological effects [3,4]. These constituents may represent potential chemical stimuli at the restoration–mucosa interface, whereas direct contact with restoration margins, surface irregularities, or roughened areas may provide a persistent low-grade mechanical irritant. This is consistent with the broader concept that dental restorative materials may be associated with localized mucosal reactions requiring careful clinicopathological interpretation [7]. From a tissue-response perspective, chemical, mechanical, and possibly electrochemical stimuli may converge on epithelial barrier stress, chronic inflammatory signaling, oxidative/redox imbalance, and reactive epithelial turnover [10,11,12,22].

A biologically plausible link between amalgam-derived metallic components and suprabasal CK19 expression may involve inflammation- and stress-mediated modulation of epithelial differentiation. Mercury-, silver-, and copper-containing phases may contribute, under conditions of restoration aging and surface degradation, to local epithelial stress responses and inflammatory activation [3,4]. Such stress may amplify inflammatory signaling within the epithelial–stromal interface, promoting cytokine-mediated basal/parabasal cell activation, increased epithelial turnover, and expansion of a regenerative epithelial compartment [8,9,10,11,12,23,24,25,26]. Because CK19 is associated with basal or progenitor-like epithelial compartments, its suprabasal redistribution in this context may reflect stress-induced regenerative plasticity and altered keratin differentiation rather than dysplastic transformation [16,19,23,24]. However, the present study did not directly quantify amalgam surface degradation, metallic ion release, tissue metal accumulation, surface roughness, galvanic currents, or downstream molecular pathways. Therefore, these mechanisms should be interpreted as biologically plausible explanatory hypotheses rather than mechanisms directly demonstrated by the present data.

Moreover, because no comparison group exposed to other long-standing restorative materials was included, the observed remodeling pattern should not be interpreted as a material-specific signature of dental amalgam. Some of the local stimuli discussed above, including marginal degradation, surface roughness, plaque retention, chronic mechanical contact, and biofilm-related irritation, may also occur adjacent to other aged restorative materials [2,4,7,12,13]. Therefore, in the present study, dental amalgam should be understood as the clinical exposure context under investigation, whereas the specificity of this remodeling phenotype for amalgam remains to be determined in future comparative studies.

CK19 redistribution was a central feature of this remodeling pattern. In normal oral epithelium, CK19 expression is generally limited to the basal cell layer, consistent with its association with progenitor-like epithelial compartments [16,23,24]. Extension of CK19 immunostaining into suprabasal layers has been frequently reported in oral epithelial dysplasia and carcinoma, where it is usually interpreted as a sign of disturbed differentiation [17,18,25]. However, this predominantly oncologic interpretation may underestimate the relevance of CK19 expression in non-neoplastic mucosal adaptation. The present findings therefore suggest that CK19 redistribution should not be regarded exclusively as a preneoplastic signal, particularly when epithelial architecture remains preserved and dysplastic features are absent.

In the present cohort, CK19 redistribution was observed in the mucosa with preserved epithelial architecture and showed close associations with both inflammatory intensity and proliferative activity. Within the correlation network, CK19 occupied an intermediate position between structural epithelial changes and functional proliferative responses, being linked to Ki67 expression and basal epithelial hyperplasia. In this context, suprabasal CK19 extension may be interpreted as compatible with enlargement of a regenerative epithelial compartment under sustained local stimulation, rather than as direct evidence of loss of differentiation control. Similar observations have been reported in inflammatory periodontal conditions, including gingivitis and chronic periodontitis, where suprabasal CK19 expression may occur without dysplasia and is commonly interpreted as a reactive change related to epithelial turnover and inflammatory activation [19,26]. These findings support the view that CK19 redistribution can accompany inflammation-driven epithelial remodeling without necessarily indicating malignant progression.

The comparison with periodontal inflammation is relevant because it emphasizes the context-dependent nature of CK19 plasticity. In chronically stimulated mucosa adjacent to amalgam restorations, suprabasal CK19 expression appears to occur within an inflammatory and regenerative background rather than within a dysplastic architectural framework. Inflammation may therefore represent one plausible upstream contributor to the remodeling pattern observed in this study, although the retrospective design does not allow direct mechanistic confirmation. The gradual increase in inflammatory infiltrate across exposure categories was not only a descriptive histological finding but also a core component of the exploratory IERS model, supporting its role in the cumulative remodeling phenotype.

Although inflammatory infiltrates increased with longer exposure duration, the structural remodeling pattern observed in this cohort cannot be attributed solely to transient inflammatory fluctuations. The coordinated progression of basal epithelial expansion, suprabasal CK19 redistribution, proliferative activity, and stromal fibrosis suggests a more stable microenvironmental adaptation rather than a purely reactive inflammatory response. This interpretation is further supported by the integrated analysis captured by the IERS composite score, which reflects the cumulative behavior of epithelial and stromal parameters rather than isolated inflammatory variation.

Chronic low-grade inflammation is known to modulate epithelial cytoskeletal organization and proliferative equilibrium through cytokine-mediated pathways and redox signaling [27]. In this setting, epithelial cells may recalibrate differentiation and regenerative dynamics to maintain barrier integrity under sustained stimulation. Ki67 labeling further contextualizes this adaptive response. Although proliferative activity increased with exposure duration, it remained within a range compatible with reactive hyperplasia rather than uncontrolled expansion. Its close association with CK19 redistribution is compatible with coordinated cytoskeletal reorganization and proliferative reinforcement, although this relationship should be interpreted as associative rather than mechanistically proven. Rather than reflecting clonal instability, this alignment is more consistent with compartmental expansion aimed at preserving epithelial resilience.

The Integrated Epithelial Remodeling Score (IERS) provided a consolidated representation of these interdependent processes. The distribution of IERS values across exposure groups indicates a generally increasing remodeling burden across longer exposure duration categories. Although the short-term exposure group (5–10 years) displayed partial overlap with control specimens, a clear upward shift was evident in the intermediate and long-term exposure categories. The continuum-like distribution supports a model of cumulative adaptation. Biological heterogeneity remained evident within each exposure group: some intermediate-duration cases approximated higher remodeling profiles, whereas certain long-exposure cases retained moderate composite values. This variability argues against a deterministic exposure–outcome mechanism and instead suggests that individual tissue responsiveness may modulate remodeling intensity. Because IERS is an exploratory composite score, it should be interpreted as a descriptive measure of remodeling burden rather than as a validated diagnostic, prognostic, or mechanistic index.

p53 expression further contextualizes this interpretation. Although p53 demonstrated statistically detectable variation across exposure categories, its distribution lacked the ordered gradient observed for CK19 and Ki67, suggesting that this signal likely reflects heterogeneous cellular stress responses rather than a primary driver of epithelial remodeling. While modest numeric increases were observed in higher exposure groups, its weaker integration within the correlation matrix indicates that p53 does not occupy a central position in the remodeling network. In the absence of dysplasia, focal or moderate p53 expression likely reflects transient stress signaling rather than mutational stabilization. However, because TP53 mutational analysis was not performed, p53 immunoreactivity should be interpreted only as a relative immunohistochemical stress-related signal and not as evidence of mutational status or premalignant transformation. The differential positioning of p53 emphasizes that not all immunophenotypic changes carry equivalent biological weight within adaptive contexts. Conceptually, the data are compatible with a framework in which chronically exposed oral mucosa may undergo regulated plastic remodeling. CK19 redistribution, basal expansion, and proliferative reinforcement appear more consistent with coordinated adaptive responses than with markers of incipient neoplasia. This distinction is particularly important given the prevailing tendency to interpret suprabasal CK19 primarily through an oncologic lens.

Contextual interpretation integrating architectural preservation, inflammatory background, and proliferative magnitude is essential. The convergence of group comparisons, correlation modeling, and composite scoring provides internal coherence to the remodeling framework described. Overall, cytokeratin redistribution and epithelial proliferative changes should be interpreted within the broader architectural and inflammatory context of oral mucosa, particularly in chronically stimulated environments where adaptive epithelial plasticity may mimic patterns traditionally associated with dysplasia.

Taken together, these findings support the interpretation of CK19 redistribution within a broader adaptive spectrum of epithelial biology. In chronically exposed yet non-neoplastic oral mucosa, cytoskeletal reorganization and proliferative modulation may represent components of structural reinforcement rather than proven mechanisms of material-induced transformation. Recognizing this adaptive dimension is critical to avoid overinterpretation of keratin patterns in contexts where architectural integrity remains intact.

### Study Limitations

Several limitations should be acknowledged when interpreting the present findings. The retrospective design and the use of archival tissue specimens limited the possibility of controlling all clinical and exposure-related variables, including oral hygiene status, local traumatic factors, dietary irritants such as frequent spicy food consumption when not consistently documented, systemic conditions, plaque-retentive factors, and other potential modifiers of mucosal inflammation. Although all included patients were documented as non-smokers in the available clinical records, smoking status was based on retrospective clinical documentation and patient-reported medical history rather than biochemical verification. Therefore, underreporting or incomplete documentation of smoking exposure cannot be entirely excluded. This is relevant because smoking may influence oral epithelial keratinization, inflammatory responses, and immunohistochemical marker expression, including CK19. Other lifestyle-related inflammatory modifiers also could not be fully assessed retrospectively. The duration of contact with amalgam restorations was derived from clinical records and was used as an indirect indicator of chronic exposure intensity. However, no direct measurement of local metallic ion release or tissue metal accumulation was performed.

An important limitation of the present study is the significant age imbalance across exposure duration groups. Patients in the longest exposure category were older than those in the shorter exposure groups, reflecting the expected clinical relationship between restoration retention time and patient age. Therefore, age may represent a relevant confounding factor, because some epithelial, inflammatory, and stromal changes may be influenced by aging-related mucosal biology independently of amalgam contact. Although age-matching would have been methodologically preferable, it was not feasible within the available retrospective archive because older control specimens fulfilling all eligibility criteria were limited. In addition, forcing strict age-matching would have reduced the control group and may have introduced selection bias related to the availability of surgically obtained control mucosa. Although exposure duration showed stronger associations with several remodeling parameters than age in the correlation analysis, and age-adjusted regression analyses were performed, these statistical approaches cannot completely eliminate residual confounding. Consequently, the present findings should be interpreted as exposure-associated remodeling patterns rather than evidence of a direct causal relationship between dental amalgam and epithelial remodeling. Future age-matched, prospectively designed, or propensity score-matched studies are required to confirm whether amalgam contact independently contributes to these mucosal changes.

Another important limitation is the absence of comparison groups exposed to other long-standing restorative materials, such as resin composites, glass ionomer cements, ceramics, or non-amalgam metallic restorations. Therefore, the remodeling phenotype observed in the present cohort cannot be considered specific to dental amalgam. Long-standing restorations, irrespective of material type, may generate local mucosal stimulation through surface aging, marginal degradation, plaque retention, surface roughness, chronic mechanical contact, or biofilm-related irritation [2,4,7,12,13]. Accordingly, the present findings should be understood as describing a remodeling pattern associated with chronic mucosal adjacency to long-standing coronal amalgam restorations, rather than as a material-specific biological signature of amalgam. Although amalgam-related chemical factors remain biologically plausible, their contribution cannot be separated from non-specific restorative-material factors or local mechanical and biofilm-related stimuli within the current retrospective design [3,4]. In addition, although exposed cases were selected based on documented adjacency or contact between the sampled mucosa and conventional coronal amalgam restorations, complete quantitative data on restoration class distribution, surface area, marginal adaptation, surface roughness, contact pressure, plaque retention, and the exact topographic relationship between the restoration and the mucosa were not consistently available. These factors may influence local mechanical, chemical, and biofilm-related stimulation and should be considered potential confounders. Future comparative and prospective studies should include different restorative materials and standardized documentation of restoration type, class, surface area, marginal quality, surface roughness, plaque retention, and mucosal contact pattern.

Although the sample size allowed the detection of statistically significant associations, the overall cohort remained relatively limited and numerically imbalanced between control and amalgam-exposed specimens. Therefore, the observed remodeling patterns should be interpreted with caution and may not fully represent the broader biological variability of oral mucosal responses to long-standing restorative materials. Larger, multicenter studies with more evenly balanced groups would be useful to confirm the reproducibility and robustness of these findings across different populations and clinical settings.

Histopathological and immunohistochemical evaluations were based on semi-quantitative scoring systems. Although such methods are commonly used in tissue-based research and were applied through independent assessment followed by consensus review, a degree of observer-related variability cannot be completely excluded. In addition, the Integrated Epithelial Remodeling Score was used as an exploratory composite measure and should not be interpreted as a validated diagnostic or prognostic tool.

Finally, the study was primarily focused on morphological and immunophenotypic characterization. Molecular analysis of inflammatory mediators, oxidative stress pathways, epithelial stemness markers, or metal-related signaling mechanisms was beyond the scope of the present investigation. Consequently, the results should be regarded as evidence of an association between chronic mucosal adjacency to long-standing amalgam restorations and coordinated non-dysplastic epithelial remodeling, rather than as direct mechanistic proof of amalgam-specific epithelial modulation. Nevertheless, the convergence of histopathological findings, immunohistochemical patterns, correlation analysis, and composite remodeling assessment provides a coherent framework supporting the concept of adaptive epithelial plasticity in chronically stimulated oral mucosa.

## 5. Conclusions

The present study indicates that prolonged contact between oral mucosa and long-standing dental amalgam restorations may be associated with a gradual, non-dysplastic remodeling response. This pattern was characterized by expansion of the basal epithelial compartment, suprabasal redistribution of CK19, increased Ki67-related proliferative activity, and persistence of a chronic inflammatory background. Importantly, these changes occurred within preserved epithelial architecture and were not accompanied by morphological evidence of dysplasia. However, the significant age imbalance among exposure duration groups limits causal interpretation. In addition, because no comparison group exposed to other restorative materials was included, the specificity of this phenotype for dental amalgam cannot be established. Therefore, these findings should be considered evidence of an exposure-associated remodeling phenotype, not definitive proof that amalgam contact independently induces epithelial remodeling. Age-matched and multivariable-controlled studies are needed to clarify the independent contribution of chronic amalgam contact to these mucosal changes.

The exploratory Integrated Epithelial Remodeling Score (IERS) further supports the interpretation that these alterations represent a coordinated remodeling phenotype rather than isolated histological or immunohistochemical variations. Within this context, CK19 redistribution appears more consistent with enlargement of a regenerative epithelial compartment than with intrinsic malignant transformation.

These findings highlight the importance of interpreting cytokeratin expression patterns in relation to tissue architecture, inflammatory status, and proliferative activity. Suprabasal CK19 expression, when observed in non-dysplastic and architecturally preserved oral mucosa, should not be automatically regarded as evidence of preneoplastic progression. Further studies incorporating molecular, inflammatory, and microenvironmental analyses are warranted to clarify the biological pathways involved in this adaptive epithelial response.

## Figures and Tables

**Table 1:** Clinicodemographic characteristics of the study cohort.

Parameter	Control Group (*n* = 30)	5–10 Years (*n* = 28)	11–20 Years (*n* = 34)	≥21 Years (*n* = 16)	*p*-Value
Age, years, mean ± SD	47.90 ± 9.39	40.54 ± 6.36	51.43 ± 6.27	62.00 ± 5.03	<0.001
Female, *n* (%)	16 (53.3)	13 (46.4)	23 (67.6)	11 (68.8)	0.084
Male, *n* (%)	14 (46.7)	15 (53.6)	11 (32.4)	5 (31.2)	0.084
Non-smokers, *n* (%)	30 (100.0)	28 (100.0)	34 (100.0)	16 (100.0)	NA
Alveoloplastic extraction, *n* (%)	30 (100.0)	28 (100.0)	34 (100.0)	16 (100.0)	NA

Note: SD = standard deviation; NA = not applicable. All specimens were obtained during alveoloplastic extractions. Data regarding frequent spicy food consumption were not consistently available in the retrospective records and were therefore not included as a comparative variable.

**Table 2:** Distribution of histopathological parameters according to amalgam exposure duration.

			History of Amalgam Exposure			*p*-Value
		0 (Control Group) (*n* = 30)	1 (5–10 Years) (*n* = 28)	2 (11–20 Years) (*n* = 34)	3 (≥21 Years) (*n* = 16)	
**Basal hyperplasia**	0	5	4	1	1	0.004
	1	16	17	12	3	
	2	9	6	16	7	
	3	0	1	5	5	
**Acanthosis**	0	0	0	0	0	0.003
	1	21	18	11	2	
	2	9	9	17	9	
	3	0	1	6	5	
**Parakeratosis**	0	28	16	16	7	0.042
	1	2	12	18	9	
**Spongiosis**	0	13	9	4	1	0.017
	1	11	13	15	5	
	2	6	5	12	7	
	3	0	1	3	3	
**Fibrosis**	1	21	17	9	2	<0.001
	2	9	9	18	7	
	3	0	2	7	7	
**Inflammation**	1	21	19	11	2	<0.001
	2	9	8	17	6	
	3	0	1	6	8	

**Table 3:** Distribution of immunohistochemical markers according to amalgam exposure duration.

			History of Amalgam Exposure			*p*-Value
		0 (Control Group) (*n* = 30)	1 (5–10 Years) (*n* = 28)	2 (11–20 Years) (*n* = 34)	3 (≥21 Years) (*n* = 16)	
**CK19**	0	9	7	2	0	<0.001
	1	16	14	13	2	
	2	4	6	14	5	
	3	1	1	5	9	
**Ki67**	0	8	6	2	0	<0.001
	1	18	18	10	2	
	2	4	4	22	14	
**p53**	0	21	20	17	4	0.023
	1	7	6	11	6	
	2	2	2	6	6	

**Table 4:** Age-adjusted regression analysis of exposure duration and remodeling outcomes.

Outcome	Model	Predictor	Effect Estimate	95% CI	*p*-Value
IERS	Multivariable linear regression	Exposure duration category	β = 1.12	0.70–1.54	<0.001
IERS	Multivariable linear regression	Age	β = 0.03	0.00–0.08	0.061
Inflammatory infiltrate ≥ 2	Binary logistic regression	Exposure duration category	aOR = 2.08	1.28–3.39	0.003
Inflammatory infiltrate ≥ 2	Binary logistic regression	Age	aOR = 1.03	0.99–1.08	0.119
Basal hyperplasia ≥ 2	Binary logistic regression	Exposure duration category	aOR = 1.82	1.14–2.91	0.012
Basal hyperplasia ≥ 2	Binary logistic regression	Age	aOR = 1.04	0.99–1.09	0.087
CK19 ≥ 2	Binary logistic regression	Exposure duration category	aOR = 2.35	1.40–3.95	0.001
CK19 ≥ 2	Binary logistic regression	Age	aOR = 1.02	0.98–1.07	0.241
Ki67 score 2	Binary logistic regression	Exposure duration category	aOR = 2.51	1.46–4.31	<0.001
Ki67 score 2	Binary logistic regression	Age	aOR = 1.03	0.98–1.08	0.168

Note: IERS = Integrated Epithelial Remodeling Score; CI = confidence interval; aOR = adjusted odds ratio. The exposure duration category was coded as 0 = control, 1 = 5–10 years, 2 = 11–20 years, and 3 = ≥21 years. Effect estimates for the exposure duration category represent the change per one-category increase. Age was entered as a continuous covariate and is reported per one-year increase. High-grade remodeling parameters were dichotomized as inflammatory infiltrate ≥ 2, basal hyperplasia ≥ 2, CK19 ≥ 2, and Ki67 score 2.

## Data Availability

Data supporting the reported results are available from the corresponding authors upon reasonable request.
